# Nature and Pattern of Cricket Injuries: The Asian Cricket Council Under-19, Elite Cup, 2013

**DOI:** 10.1371/journal.pone.0100028

**Published:** 2014-06-13

**Authors:** Nabangshu S. Das, Juliana Usman, Dipankar Choudhury, Noor Azuan Abu Osman

**Affiliations:** 1 Department of Biomedical Engineering, Faculty of Engineering, University of Malaya, Kuala Lumpur, Malaysia; 2 Centre of Applied Biomechanics, University of Malaya, Kuala Lumpur, Malaysia; 3 Faculty of Mechanical Engineering, Brno University of Technology, Brno, Czech Republic; 4 CEITEC-Central European Institute of Technology, Brno University of Technology, Brno, Czech Republic; University of New South Wales, Australia

## Abstract

Cricket has over the years gained much popularity in Asia, thus the number of cricket players has also grown in tandem. However, cricket players are not as fortunate as other athletes as they do not always have a standard cricket infrastructure to practice; therefore, the injury prevalence is expected to be high. Unfortunately, very few studies have been conducted to investigate the nature and pattern of cricket injuries prevalent to cricketers in this region. Therefore, a prospective cohort injury surveillance study was conducted during the Asian Cricket Council (ACC) Under-19 Elite Cup held in June 2013 in order to gather more data on the type of injuries sustained by cricket players. Overall, 31 injuries occurred to 28 players throughout the tournament, of which 7 injuries happened during practice sessions. The overall injury incidence rate (IIR) was 292.0 per 10,000 player hours (95% CI 176.9–407.1) and 10.4 per 10,000 balls faced and 2.6 per 1000 overs bowled delivered during batting and bowling, respectively. Injuries to the lower limb (IIR: 146; 95% CI 1.8–98.2) were the most frequent, followed by injuries to the upper limb (97.3;95% CI 30.2–164.5) and to the trunk and back (IIR: 36.5;95% CI 0.0–77.7). Sprain/strains (IIR 109.5;95% CI 38.4–180.7) to muscle/tendon and joint/ligament were the most commonly reported nature of injury.

This is the first study investigating injury incidence among the players of the ACC. It provides an overview of injuries sustained by elite players' under-19 years of age from10 Asian countries. The overall IIR is similar to earlier studies conducted in well-established cricket playing nations.

## Introduction

Since the introduction of the First Limited Overs cricket in 1963, the sport has become more vibrant, and currently cricket is one of the most popular played sports in Australia, England, in the Indian subcontinent, the West Indies and South Africa. Recently, the International Cricket Council (ICC) has taken an initiative to expand the popularity of cricket among their associate member nations; Afghanistan, Bahrain, Hong Kong, Kuwait, Qatar, Malaysia, Nepal, United Arab Emirates(UAE), Saudi Arabia, and Thailand [1]. The Asian Cricket Council Under-19, Elite Cup tournament arranged by the Asian Cricket Council (ACC) is a part of this initiative [2]. The cricket infrastructure in these countries are still developing, therefore, injury surveillance is essential for identifying the potential injury risk factors in order to develop a mechanism for effective injury prevention [3]. A consensus for injury surveillance method in cricket has been established in 2005 [4]. Following the establishment of the consensus, the overall injury incidence rate (IIR) in One Day International (ODI) were reported to be 32.1, 51.6, 40.6 and 90/10,000 player hours in Australia [5], [6], New Zealand [7], South Africa [8] and West Indies [9], respectively. Bowlers were found to be most susceptible to the injury [6]–[9]. Dhillon et al. [10], conducted the only study known from an Asian country which was performed in India. However, the study focused only on upper limb injuries and they found that most of the injuries; 10 out of 16 injuries occurred during fielding. Injury incidence rates were found to be higher at the international game than on the domestic format [7], [9]. These studies reported only time-loss injuries, which corresponds to the consensus of international injury definition in cricket [4].

In a short tournament, it is possible to count accurately both time-loss and non-time loss injuries. During the last ICC World Cup, 2011, both time-loss and non-time loss injuries were considered, resulting in a much higher overall IIR of 314.3/10,000 player hours with bowling match injuries amounting to 7.5/1000 overs bowled and batting match injuries recorded at 17.8/10,000 balls faced [11]. The data were obtained from five of the fourteen participating nations [11]. To our knowledge, no cricket surveillance has been conducted focusing on the cricketers from associate members of the ICC. Therefore, the objectives of this study were to analyze the collected data on cricket injury during the ACC U-19 Elite Cup, 2013, and determine the potential risk factors. The data collected would serve as an important injury data bank in Asian cricket, which could motivate the ACC management to conduct further studies into this area. This will provide effective prevention and management of cricket sport injuries in the Asian region.

## Method

### Ethics Statement

The study was conducted during the ACC U-19, Elite Cup 2013 which was held in Kuala Lumpur, Malaysia. All participants were male players from the ten Asian associated members of the ICC, and all provided written informed consent. On behalf of the junior players (those under 18 years old) enrolled in the study, written informed consent was also acquired from the management committee for the Asian Cricket Council (ACC) Under-19 Elite Cup 2013. Parents and/or guardians of the junior players were not approached for their written informed consent as the players came from 10 Asian countries and travelled to the tournament country with their management team as their guardians. As such it was only feasible to get written informed consent from the management committee for the ACC Under-19 Elite Cup 2013. All data used in this study was analysed anonymously. The study was approved by the research committee of the University of Malaya. The ethics approval included both the junior cricket players aged 18 and below and the senior cricket players aged above 18 years old.

The injury definition used in this study was any injury or medical condition which was sustained during play in a match which prevented a player from being fully available either during a match or for selection for a major match [4]. It is to be mentioned here that both time-loss and non-time loss injuries were taken into account. If an injury resulted in the player being unavailable for selection, it was considered as a time-loss injury, while non-time loss injury was an injury that did not render player unavailable for selection at any time during the tournament. Standard injury report form was used to collect injury information which includes the type of injury, nature of injury, anatomical injury location, playing position, and the injury mechanisms. The main outcome measure was the injury incidence rate (IIR) which included the injury incidence rate per 10,000 player hours, the injury incidence rate per 10,000 balls faced, and the injury incidence rate per 1000 overs bowled. The injury incidence rate per 10,000 balls faced and the injury incidence rate per 1000 overs bowled indicated the specific play of batting and bowling, respectively. It is to be noted that there are 6 balls per overs. In this study, only match exposure hours were calculated. Calculation for injury incidence rates followed the guidelines as published by Orchard et al. (2005) [4]. The number of games per team played was recorded during the study period to help calculate player exposures. Injury rates and the 95th percentile confidence intervals were then calculated. Poisson regression analysis was conducted to study the relationships between the following factors and injury: age, Body Mass Index (BMI), and playing experiences. Alpha was set at 0.05. All statistical analyses were conducted with SPSS version 20.

## Results

A total of 129 players were recruited from a potential pool of 140 participants prior to the start of the tournament. However, only 112 players were included in the final analysis due to unavailability of a permanent physiotherapist to help provide professional injury assessment in one of the team and the failure of another team to record and return the injury report forms given.

With 112 participants, a total of 821.9 player participating hours were recorded throughout the tournament covering 27 matches. The mean age of the players were 17.1±1.6 years old, height 173.1±7.6 cm, body weight 66.5±10.5 kg, BMI 22.3±3.9 kgm^−2^, and playing experiences were recorded at 7.7±3.0 years. The pre-season survey showed that 48.8% of the participants had experienced an injury within the previous 5 years (2009–2013) of playing and the top 4 injuries sustained among players were: fingers (24.2%), shoulder (15.9%), and ankle and back (11.3%) respectively.

Overall, 31 injuries sustained by 28 players were reported throughout the tournament, of which 7 injuries occurred during the practice sessions. However, the 7 recorded practice injuries were excluded from further analysis since it was not possible to determine the total exposure hours at practice sessions. More than 90% (n = 22) of the total match injuries were new and the remaining 8.3% were recurrent injuries. The overall injury incidence rate was 292.0 per 10,000 player hours (95% CI 176.9–407.1). There was no significant relationship found between the IIR and the demographic characteristics; age (p>0.05), BMI (p>0.05) and experiences (p>0.05). Injuries to the lower limb (IIR: 146.0; 95% CI 1.8–98.2) were the most common, followed by injuries to the upper limb IIR 97.3 per 10,000 player hours (95% CI 30.2–164.5) and the trunk and back IIR: 36.5 (95% CI 0.0–77.7) (Table-1). Of the injuries that occurred to the lower limb, most (n = 7, 58.3%) were acquired by the batsmen followed by the fielders (n = 4, 33.3%). In contrast, of the injuries that occurred to the upper limb, the fielders sustained the highest injury incidence (n = 5, 45.4%) and this was followed by the wicket keepers (n = 2, 25.0%).

**Table 1:** Susceptible body region to injury.

Body Region	n	IIR/10000 player hours	95% CI Value	Activities			
				Batting, n (%)	Bowling, n (%)	Fielding, n (%)	Wicket keeping, n (%)
**Upper Limb**	**8**	97.3	30.2–164.5	1 (11.1)	0	5 (45.4)	2 (100.0)
* Shoulder*	*1*						
* Upper arm*	*3*						
* Forearm*	*1*						
* Hand/finger*	*3*						
**Trunk and Back**	**3**	36.5	0.0–77.7	1 (11.1)	0	2 (18.2)	0
* Lower back*	*1*						
* Hip*	*1*						
* Groin*	*1*						
**Lower Limbs**	**12**	146	1.8–98.2	7 (77.8)	1 (50%)	4 (36.4)	0
* Thigh*	*4*						
* Knee*	*1*						
* Lower leg*	*4*						
* Ankle*	*1*						
* Foot/toes*	*2*						
**Others**	**1**	12.2	0.0–36.1	0	1 (50%)	0	0
Total	24	292.0		9 (100.0)	2 (100.0)	11 (100.0)	2 (100.0)

* *The percentage values were calculated against the total number of injuries in each activity.*

The injury incidence rates during specific play of batting and bowling were recorded to be 10.4 injuries per 10,000 balls and 2.6 injuries per 1000 overs bowled, respectively. Table 2 shows the injuries based on player position and their mechanisms of injury. Of the injuries that occurred, the highest proportion was reported to have occurred during fielding (n = 11, 45.8%) followed by batting (n = 8, 33.3%) and bowling (n = 2, 8.3%). Non-contact activities i.e. throwing, running, after bowling delivery had accounted for 40.9% of the total injuries.

**Table 2:** Injuries according to role in the match.

Mechanisms by role in match	N	Percentage (%)
**Batting**	**8**	33.3
*Running*	*3*	
*Stuck by ball*	*2*	
*Unknown*	*3*	
**Wicket keeping**	**3**	12.5
*Throwing*	*2*	
*Stuck by ball*	*1*	
**Fielding in total**	**11**	45.8
*Sliding*	*1*	
*Throwing*	*4*	
*Catching*	*4*	
*Unknown*	*2*	
**Bowling**	**2**	8.3
*After delivery*	*2*	
**Total**	24	100.0

Sprain/strain (IIR 109.5; 95%CI 38.4–180.7) were the most commonly reported injury followed by inflammation/swelling (IIR: 48.7; 95%CI 1.1–96.2) (Table- 3).

**Table 3:** Nature of injury.

Nature of Injury	n	Percentage (%)	IIR/10000 hours	95% CI value
Inflammation/Swelling	4	16.6	48.7	1.1 – 96.2
Bruise/Contusion	1	4.2	12.2	0.0 – 36.0
Strain/Sprain	9	37.5	109.5	38.4 – 180.7
Concussion	1	4.2	12.2	0.0 – 35.9
Superficial*	1	4.2	12.2	0.0 – 35.9
DOMS	1	4.2	12.2	0.0 – 35.9
Impingement	1	4.2	12.2	0.0 – 35.9
Cramping	2	8.3	24.3	0.0 – 58.0
Others**	4	16.6	48.7	1.1 – 96.2
**Total**	24	100.0	292.0	– 407.1

* *Lesion on skin.*

** *Injuries which were not mentioned by the sports physiotherapists and the players.*

## Discussion

The strength of this study is that it has covered and recorded the IIR at an international junior tournament among the Asian associated members where at the moment, there is no or little information known on injury rates within this cohort. This data is important and of significance as the ICC is particularly focusing on further development in this area [12]. Results of the current study would encourage the ACC governing body to arrange a more rigorous investigation into the cricket injury rate as well as performance development. However, the tournament period was quite short (two weeks), and the injury patterns may vary from a longer full session studies.

The overall match injury incidence rate was 292.0 injuries per 10,000 player hours, which was similar to the incidence reported on Cricket World Cup 2011; 314.3 injuries per 10,000 match hours [11]. Most of the injuries in the present study occurred during match period, this is in agreement with earlier studies done to assess cricket injury incidence rate [9], [13].

Recent trend has shown equal distribution of injuries across batting, bowling and fielding at junior level [13] while at the higher performance playing level, bowling contributed up to twice the number of injuries sustained compared to batting and fielding as conducted by Orchard et al [6]. In contrast, in this study, fielding was accounted for around half (45.8%) of the total injuries. It is worth noting that in previous studies [6], [13] the collected injury data were from three full playing seasons, while our study was based on a shorter version of a normal tournament. The most amount of fielding injuries occurred during catching and throwing balls which indicates a lack of skill among the players. Ground hardness might have also played a role as most of the injuries sustained were during catching, sliding, and running. There has been an association between ground hardness and injuries due to being struck by ball, mishandling of the ball while fielding, diving for a catch or slip/trip [14].

In this study, injuries caused by being struck by a ball was found to be the highest and this often appears to be the single most reason for injury at the junior level cricket. These findings are in agreement with the study done by Finch et al. (2010) [13].

The lower limb was the most vulnerable body region to get injured at the junior and professional level of play [13], [6]. In the present study, 50% of the total injuries were to the lower limb while 77.8% of those injuries were sustained during batting. However, in the case of fielding, half of the reported injuries were to the upper limb and were associated with catching and throwing which are classified as contact injuries. Wearing appropriate protective equipment might help in preventing and minimizing contact injuries. However, further investigation in this area needs to be carried out to gauge the effectiveness of protective equipment in injury prevention among cricket players especially at the junior level. In the case of non-contact injury, it is suggested that the players improve their skill and fitness through appropriate training. In contrast with previous studies[11], [15] where bruising was reported as the most common nature of injury, the present study revealed that strain/sprain as the most common type of injury. Such injury was uniformly distributed among batting, bowling and fielding activities.

Previous studies indicated that fast bowlers were exposed to high rate of severe overuse injury [16]–[18]. Coaching intervention studies had been carried out to correct the techniques in bowling actions and were reported to make a contribution in the reduction of low back injury to the bowlers [19], [20]. Wallis et al. (2002) suggested a coaching intervention involving a wearable bowling harness (brace) in order to restrict the movement of the shoulders during the delivery stride which showed a significant improvement in decreasing the ‘twist’ experienced in the trunk during bowling [21]. This suggested that applying known coaching interventions in junior cricketers should be considered to prevent injury at the early level of play.

There has been no published information on injury investigation during the ACC U-19, Elite Cup which is held every two years since the year 1997 [22]. This study is the first done to investigate injury incidence among the players of the ACC. It provides an overview of injuries sustained by elite players' under-19, from 10 Asian counties. However, the result from this study is not a representative of all children playing cricket. A study by Finch et al. (2010) on U12, U14, and U16 junior players suggested that the rate of injury increased with increasing age level of play [13]. It is recommended that further investigation into the epidemiology of injuries among the Asian senior and junior players is be carried out in a larger cohort for a more comprehensive and inclusive outcome.

One of the limitations of this study was the small number of participants involved and the short time duration of the tournament. Information on the physical conditions of players following an injury was not collected. Therefore, severity of the incidence could not be evaluated. Taking only the match exposure time was another limitation of the study. It is suggested that the duration of practice exposure should be taken as well to evaluate the impact of practice on their performance.

## Conclusion

This is the first study in its kind done to i investigate injury incidences among the players of the ACC. It provides an overview of the injuries sustained by elite players' under-19 years of age among 10 Asian countries. The overall IIR is similar to the findings of earlier studies done in well-established cricket playing nations. Since there are no established or known publications on injury reports for the participating countries it is not possible to make a meaningful comparison to the existing result.
